# Algorithm and Software to Type Stx Operons Accurately from Assembled Genomic Sequence

**DOI:** 10.3390/microorganisms14081607

**Published:** 2026-07-23

**Authors:** Arjun Balmiki Prasad, Stephanie Abromaitis, Vyacheslav Brover, Michael Feldgarden, Katrine Grimstrup Joensen, Curtis James Kapsak, Rebecca L. Lindsey, Linlin Li, Valeria Michelacci, Susanne Schjørring, Flemming Scheutz, William Klimke

**Affiliations:** 1 National Center for Biotechnology Information, National Library of Medicine, National Institutes of Health, Bethesda, MD 20892, USA; brovervv@ncbi.nlm.nih.gov (V.B.); michael.feldgarden@gmail.com (M.F.); klimke@nih.gov (W.K.); 2 Microbial Diseases Laboratory, Center for Laboratory Sciences, California Department of Public Health, Richmond, CA 94804, USA; stephanie.abromaitis@cdph.ca.gov (S.A.); linlin.li@cdph.ca.gov (L.L.); 3 Department of Bacteria, Parasites & Fungi, Statens Serum Institute, 2300 Copenhagen, Denmark; knj@ssi.dk (K.G.J.); ssc@ssi.dk (S.S.); 4 Theiagen Genomics, Highlands Ranch, CO 80129, USA; kapsakcj@gmail.com; 5 Enteric Diseases Laboratory Branch, U.S. Centers for Disease Control and Prevention, Atlanta, GA 30329, USA; 6 European Reference Laboratory for *E. coli* Including Verotoxigenic *E. coli* (VTEC), Istituto Superiore di Sanità, 00161 Rome, Italy; valeria.michelacci@iss.it; 7 The International Escherichia and Klebsiella Centre, Section of Foodborne Infections, Department of Bacteria, Parasites and Fungi, Statens Serum Institut, 2300 Copenhagen, Denmark

**Keywords:** Shiga toxin (Stx), Shiga toxin-producing *Escherichia coli* (STEC), whole genome sequencing (WGS), pathogen surveillance, hemolytic uremic syndrome (HUS), AMRFinderPlus, Stx typing algorithm

## Abstract

Shiga toxins in Shiga toxin-producing *Escherichia coli* (STEC) infections are responsible for bloody diarrhea and serious complications such as hemolytic uremic syndrome. Two types of toxins have been identified: Shiga toxin type 1 (Stx1) and the immunologically distinct Shiga toxin type 2 (Stx2). Numerous STEC that express toxin variants within those two major groups have been characterized, some of which confer unique biological properties. These variants are grouped within the Stx1 or Stx2 types and are often assigned subtypes to indicate they are not identical in sequence or phenotype. Because serious outcomes of infection are associated with certain Stx subtypes, there is a need to assign Stx sequences to the proper subtype. Here, we report a comprehensive analysis of known Stx subtypes and describe a scheme and algorithm to classify the Stx toxins and Stx operon sequences by phylogenetic sequence-based relatedness of the holotoxin conforming to historical type designations. We used this analysis to develop the free and open-source StxTyper software and database that implements this typing algorithm; StxTyper is also integrated into AMRFinderPlus 4.0 at the National Center for Biotechnology Information (NCBI). We validated and compared the results to PCR assays on a set of isolates and current state of the art surveillance methods used in the Danish public health system, and we summarize StxTyper results for over 111,000 publicly available *E. coli* genomes. We further propose a procedure to coordinate naming and identification for newly discovered and characterized Stx subtypes.

## 1. Introduction

Shiga toxin (Stx) is produced by Shiga toxin-producing *Escherichia coli* (STEC), a major cause of gastrointestinal illnesses, leading to an estimated 357,000 cases per year in the U.S. [1]. STEC infection can result in severe disease such as hemolytic uremic syndrome (HUS), which may lead to hospitalization and death. Shiga toxin itself is made up of two subunits, A and B, and the mature holotoxin has a polypeptide structure of five B subunits to one A subunit. The toxins all bind to specific glycolipid receptors and share biological properties, including enterotoxicity in ligated rabbit ileal loops, neurotoxicity in mice, and cytotoxicity to receptor-expressing cell lines such as Vero and HeLa cells.

The genes that code for Shiga toxin, *stxA* and *stxB*, occur in an operon separated by a short spacer sequence carried on a phage that can transmit the genes from one bacterium to another. The toxins coded for by these operons are categorized into types Stx1 and Stx2, with subtypes of each designated by trailing letters. Stx1 and Stx2 diverge by 50–60%, with Stx2 being more divergent and more often associated with severe disease. The different subtypes have different phenotypic behaviors and are associated with different in vitro phenotypes, disease-related characteristics, and hosts of the bacteria [2,3]. At the time of this paper, there have been four subtypes of Stx1 (a, c, d, e) and 15 subtypes of Stx2 (a–o) described [4,5,6,7].

Consistent and accurate subtype nomenclature is essential for public health surveillance, outbreak investigation, and predicting the clinical risks associated with STEC infections and STEC reservoirs in the natural world [8]. The increasingly routine use of whole-genome sequencing (WGS) in surveillance and treatment has led to a large influx of STEC isolate assemblies submitted to International Nucleotide Sequence Database Collaboration (INSDC) databases [9]. While this data is invaluable for tracking pathogens, the scale makes manual determination of Stx subtypes impractical. Unfortunately, existing automated solutions may be incomplete or suggest incorrect types for novel operon sequences [10,11].

Previous analysis of *E. coli* genomes found in NCBI’s Pathogen Detection System, which includes public isolate sequence data for *E. coli* including STEC and other pathogens, identified novel Shiga toxin (Stx) operons [5]. As a result of that work, we surveyed the landscape of *stx* genes and analyzed known Stx operon sequences. Here, we describe that analysis, along with the development and testing of an algorithm and software, to determine Stx subtypes from assembled nucleotide sequence. This approach standardizes the sequence-based assignment of subtype for the Shiga toxins, preserves historical subtype designations that are based both on differences in biological properties of the toxins and relatedness, and makes subtypes easily predictable from sequence. Based on that work, we developed the StxTyper software and database to implement the typing scheme. We validated this method using sets of STEC genomes previously typed using established PCR assays and methods based on whole-genome shotgun sequencing. We demonstrate the power of this new tool by applying it to over 111,000 whole-genome sequenced STEC isolate assemblies with full or partial *stx* genes and operons using the NCBI Pathogen Detection System [https://www.ncbi.nlm.nih.gov/pathogens accessed on 2 April 2025]. We further propose a procedure to identify and name new subtypes as they are discovered and characterized. The StxTyper software and database are publicly available independently at https://github.com/ncbi/stxtyper and as part of AMRFinderPlus (https://github.com/ncbi/amr; [12]).

## 2. Materials and Methods

### 2.1. Stx Operon Analysis and Algorithm Development

As described in Lindsey et al., 2023 [5] we identified *stx*-related sequences in GenBank using NCBI Pathogen Detection data. From that set we collected nucleotide sequences of the full *stx* operon that encode the signal peptides (66 bp in the A subunit, and 60 and 57 bp respectively in the B subunits of *stx1* and *stx2*), the A subunit (879 bp in *stx1* and 891 bp in *stx2*), the intergenic region (9–12 bp) and the B subunit (207 bp for *stx1* and 204 or 210 bp for *stx2*), as well as the amino acid (AA) sequences for the combined A and B holotoxin. Translated sequences from the open reading frames, predicted by sequences to encode the holotoxin A and B subunit sequences, were imported into a BioNumerics v8.1 database (Applied Maths, bioMérieux, Marcy-l’Étoile, France).

The holotoxin AA sequences of Stx1 and Stx2 were analyzed separately and compared by unweighted pair group method using arithmetic averages (UPGMA), with an open gap penalty of 100%, a unit gap penalty of 0%, the fast algorithm at a minimum match sequence of 2, and a maximum number of gaps of 10, followed by multiple alignments and creation of a consensus sequence from the root of the obtained dendrogram. Neighbor-joining cluster analysis with the same algorithm as for UPGMA was used to analyze the global cluster calculations. Evolutionary unrooted trees were created from maximum parsimony cluster analysis using 100 bootstrap samples. In addition, the AA sequences were analyzed for sequence motifs that would support the phylogenetic analyses.

The full nucleotide sequences, including the intergenic region, were analyzed by the same procedure to evaluate the possible differences between nucleotide and AA sequences. We excluded partial sequences from the analysis in the assignment of subtype designations. Discrepancies between the neighbor-joining and the maximum parsimony cluster analysis of the AA sequences were resolved using the evolutionary unrooted tree from maximum parsimony and compared to nucleotide analyses, in order to assign subtypes and variants. The sequence for *S. dysenteriae 1* strain 3818T (Accession No. M19437 [13]) was used as the reference sequence for analysis of Stx1. The sequence for O157:H7 strain EDL933 (Accession No. X07865 [8]) was used as the reference sequence for analysis of Stx2. Partial sequences were excluded from the analyses in the assignment of variant designations. A variant was defined by one AA difference in the analyzed sequences compared to the other sequences. The first valid published sequence was chosen to represent each specific variant. See Section 3.3 *Typing Algorithm* below for the full description of the resulting algorithm.

### 2.2. StxTyper and Operon Detection

StxTyper (https://github.com/ncbi/stxtyper) uses translated BLAST [14] alignments to a reference database of StxA and StxB protein sequences that is included with StxTyper and can be found in the NCBI Pathogen Detection Reference Gene Catalog (https://www.ncbi.nlm.nih.gov/pathogens/refgene accessed on 25 March 2025). Alignments are screened according to the following algorithm to determine whether they are full-length, and only complete operons are subtyped (e.g., Stx1c, Stx2n). Those that are not complete operons or cannot be fully subtyped by the scheme will be assigned Stx types of Stx1 or Stx2, or if indeterminate, Stx.

#### Algorithm for Determining If Operons Are Complete

StxTyper uses the following algorithm to identify putatively incomplete or non-functional Stx operons. These categories are included in the “operon” field of StxTyper and also appear in the AMRFinderPlus “Method” field when run by AMRFinderPlus version 4.0 or later [12,15].

Alignments or operons that are <80% identical to individual reference subunits or <80% identical to both A and B subunits combined are dropped from consideration to avoid false positives, but also enable the detection of novel subunits. Subunits A and B are required to be in the correct order and orientation. From the remaining alignments, the putatively non-functional categories are determined in this order:

1. FRAME_SHIFT is when there are two consecutive BLAST hits to the same reference with distance < 10 bp in different reading frames. Note that because these blast matches to the whole reference protein are split into multiple alignments, the numbers for percent identity and alignment length are calculated from the identified alignments and may differ from expectations.
2. INTERNAL_STOP is called when there is a ‘*’ character (stop codon) at any place in the query portion of the alignment other than at the last position of the reference sequence.
3. PARTIAL_CONTIG_END is used to differentiate operons that may be split by assembly or sequencing issues at the end of a contig, versus PARTIAL operons internal to contigs that are more likely to be incomplete in the source genome. PARTIAL_CONTIG_END operons are identified by alignments that terminate internal to the reference sequences < 3 bp from the end of a contig and thus might be full length sequences in a higher quality assembly or sequence. A single-subunit operon is also identified as PARTIAL_CONTIG_END if the length of the unmatched portion of the contig in the same orientation as the missing subunit is <36 bp (maximum allowed intergenic region) + 60 bp (minimum coding length to determine a subunit alignment has been missed) from the end of the contig.
4. EXTENDED is when the whole reference protein aligns, but the stop codon is not present at the end of the alignment to the reference sequence.
5. PARTIAL is for the remaining cases where <100% of the reference sequences of both subunits align or the A and B subunits are >36 bp apart or have incorrect order or orientation.

Operons that do not meet any of these characteristics are designated either COMPLETE, COMPLETE_NOVEL, or AMBIGUOUS and, where possible, given the complete operon typing scheme described below, typed down to the subtype (e.g., stx2a). Stx operons that are putatively incomplete or non-functional are assigned to the type level (i.e., stx1 or stx2) if possible.

### 2.3. Datasets and Comparison of Results

#### 2.3.1. Danish Public Health Surveillance Dataset

A total of 2486 Danish Shiga toxin-producing *E. coli* (STEC) isolates, collected between 2015 and 2024 as part of national public health surveillance, were sequenced using Illumina technology. Raw reads were deposited in the Sequence Read Archive (SRA), and the corresponding NCBI Pathogen Detection assemblies were submitted to GenBank (accessions listed in Supplementary Table S1).

The Danish surveillance system relied on a combination of typing methods, which were continuously adapted over time. From 2015 to 2017, *stx*-subtypes were determined by submitting FASTQ files to the VirulenceFinder 1.7 web tool [11,16]. From 2017 onwards, *stx* identification and subtyping were performed using a combination of assembly-based and read-mapping approaches. The assembly-based analysis was initially conducted with the *E. coli* plugin in BioNumerics (Applied Maths, bioMérieux, Marcy-l’Étoile, France), which relies on SPAdes assemblies and was originally based on the VirulenceFinder database. This plugin was subsequently expanded to include additional genes, and an in silico PCR module was later incorporated. In parallel, *stx* detection and subtyping were carried out using the same database with the KMA mapping method [17]. In selected cases—particularly where multiple *stx2* subtypes were suspected—additional mapping analyses were performed in CLC Genomics Workbench (CLC bio) using the VirulenceFinder database.

Results from both the assembly-based plugin and the mapping approaches were manually compared to obtain the final subtype calls. In cases of unresolved ambiguity, *Stx* subtype determination was finalized in the laboratory using conventional PCR as described by Scheutz et al., 2012 [18].

#### 2.3.2. Real-Time PCR Dataset from the California Department of Public Health (CDPH)

A total of 523 randomly selected routine surveillance isolates received in 2023 by the California Department of Public Health Microbial Diseases Laboratory (CDPH-MDL) were analyzed for this study. The CDPH-MDL STEC surveillance workflow included PCR confirmation of the *stx* gene using primers and probes designed by the Wadsworth Center, New York State Department of Health, to detect *stx1* and *stx2* [19]. PCR was performed as described by Patterson et al., 2026 [20]. Isolates that were PCR positive for *stx* were then sequenced for state and national surveillance using Illumina technology. The whole-genome shotgun sequences were deposited in the Sequence Read Archive (SRA and NCBI Pathogen Detection assemblies were submitted to GenBank. Accessions in Supplementary Table S2).

#### 2.3.3. NCBI Pathogen Detection WGS Assemblies

Data were collected from the NCBI Microbial Browser for Genetic and Genomic Elements (MicroBIGG-E) for all *E. coli* and *Shigella* isolates that contained *stx* genes, identified by AMRFinderPlus on 3 April 2025, in isolates sequenced and submitted to INSDC databases as SRA reads or whole-genome assemblies. This set includes all assemblies analyzed in the previous two datasets. Accessions and data from this analysis are available in Supplementary Tables S3 and S4.

## 3. Results

### 3.1. Survey of Publicly Available Stx Sequences

We first surveyed the available *stx*-related sequences in GenBank and the NCBI Pathogen Detection System and collected nucleotide sequences of the full *stx* operon that included the signal peptide, the A subunit, the intergenic region, and the B subunit, as well as the amino acid (AA) sequences for the combined A and B holotoxin, as described in Lindsey et al., 2023 [5]. Variants defined as unique AA sequences for the two subunits were identified and classified by alignment and comparison to reference sequences (Supplementary Table S5 lists the analyzed variants and their accessions). The holotoxin AA sequences of Stx1 and Stx2 were analyzed separately and compared by UPGMA and neighbor-joining cluster analysis to inform the global cluster calculations. The AA sequences were analyzed for sequence motifs that would support the phylogenetic analysis. The full nucleotide sequences, including the intergenic region, were analyzed by the same procedure to evaluate the possible differences between nucleotide and AA sequences.

**Figure 1:**
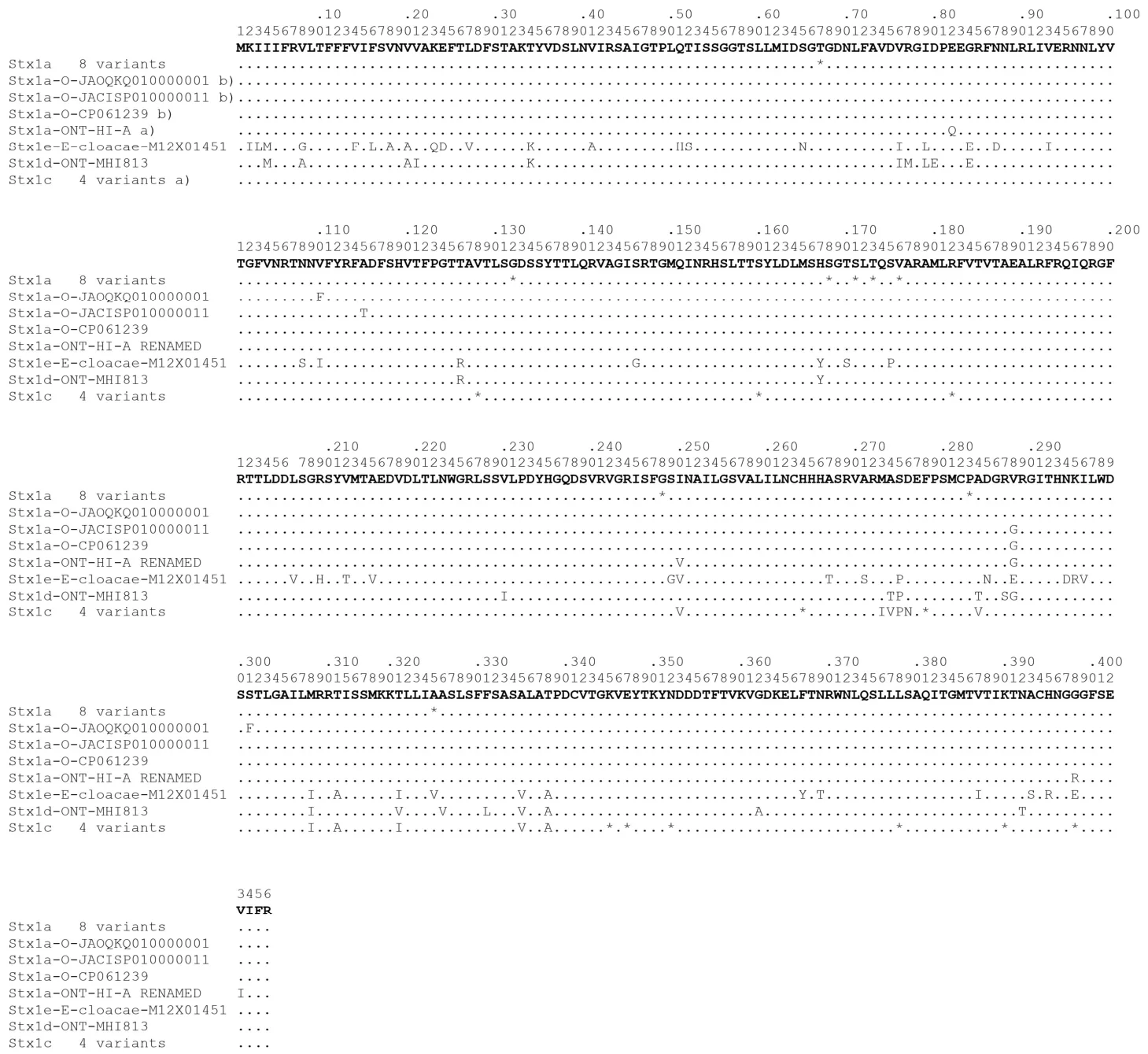
Amino acid sequences of the complete Stx1 holotoxins including the signal peptide regions and the complete A- and B-subunits and mature toxins of the 18 variants within the four subtypes of Stx1 (accessions listed in Supplementary Table S5). *: Minor single substitutions in one or more variants within each of the 4 subtypes. a) Stx1c variants comprise four distinct sequences. b) Stx1a variants comprise three subgroups with 8 variants.

**Figure 2:**
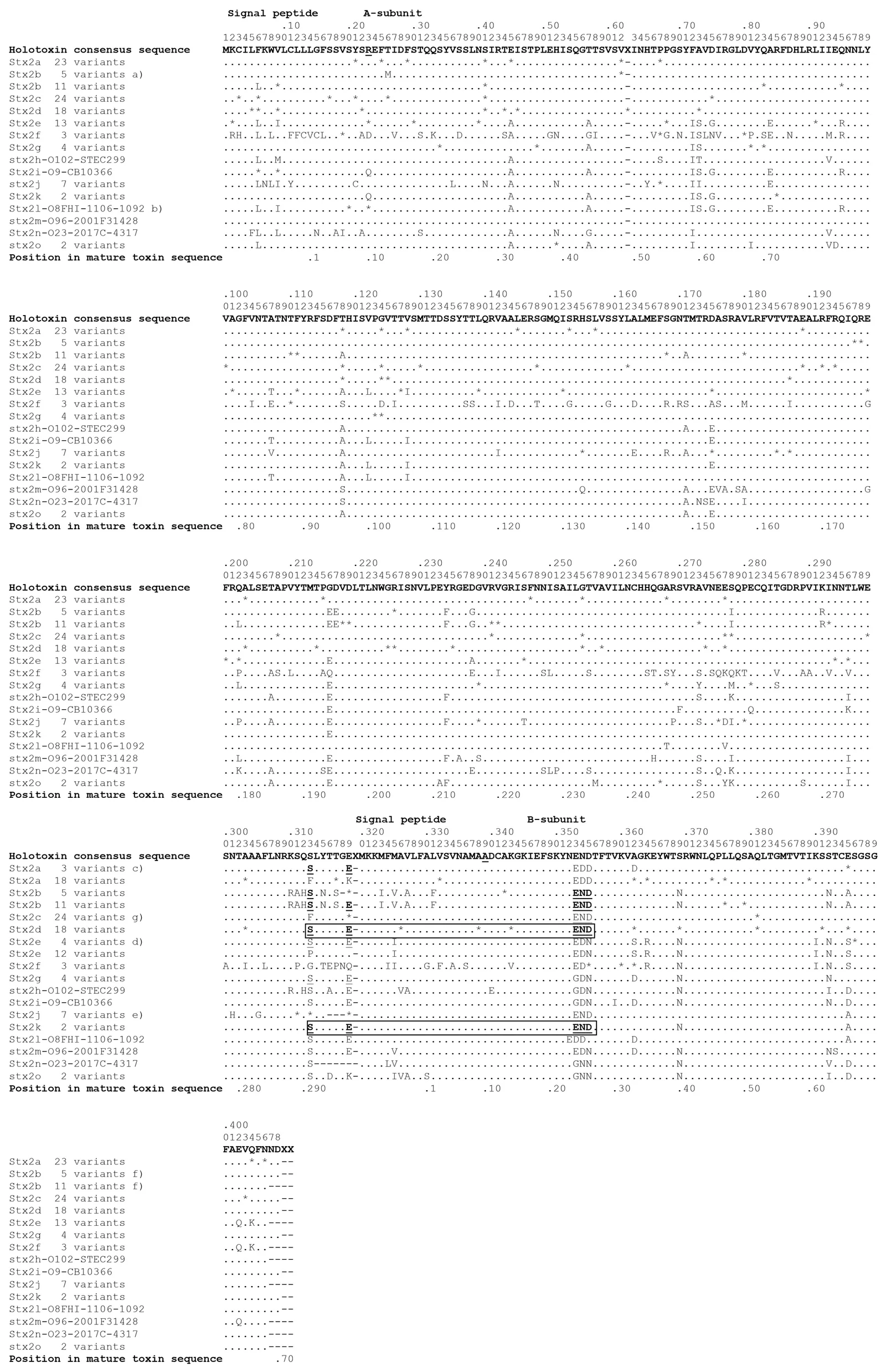
Amino acid sequences of the complete Stx2 holotoxins (top numbers) including the signal peptide regions and the complete A- and B-subunits and mature toxins (bottom numbers) of the 117 variants within the 15 subtypes of Stx2. Accessions listed in Supplementary Table S2. *: Minor single substitutions in one or more variants within each of the 15 subtypes. The first AA in the mature holotoxin consensus sequence of subunits A and B are underlined. a) Stx2b variants comprise two subgroups with five variants (Stx2b-ONT-I7606, Stx2b-O111-PH, Stx2b-O174-031, Stx2b-O128-24196-97 and the new variant Stx2b-O146-BMH-17-0027 in one group and the other 11 variants in the other group. b) Stx2e-O8-FHI-1106-1092 has been re-named to Stx2l-O8FHI-1106-1092. A similar variant (Stx2l-O65-1610T27873, unpublished) was found in a Danish patient in 2010. c) A subgroup of three Stx2a variants (Stx2a-O8-BMH-17-0027, Stx2a-O104-G5506 and Stx2a-O8-VTB178) differs from 21 other Stx2a variants by S and E in positions 313 and 319. d) Only four Stx2e variants (Stx2e-OR-TS09-07, Stx2e-O26-R107, Stx2e-O139-S1191 and Stx2e-O101-E-D43) have the S in position 313. e) The seven Stx2j subtypes have a 12 nucleotide deletion toward the end of 3’end of subunit A. f) Seven Stx2b variants lack the last two amino acids, asparagine (N) and aspartic acid (D). g) One new Stx2c variant (Acc. No. WSHT01000077) has an E at pos.

The following steps were taken to identify new variants within the subtypes:

1. The full-length protein sequences of subunits A and B were extracted and then concatenated.
2. For Stx1 subtypes, the protein identity thresholds described in section “Stx1” were used (See also Table 1 and Figure 1).
3. For Stx2a-Stx2e subtypes, the positions below correspond to the positions in the consensus holotype in Figure 2 and were used to distinguish subtypes stx2a-Stx2e, note identity thresholds are not sufficient to discriminate between Stx2 subtypes (Table 2). See Table 3 for signature residues in the Stx2 holotype alignment.
4. For non-Stx2a-Stx2e subtypes, the concatenated sequences were compared to the reference alignment provided in section “Stx2”.

#### 3.1.1. Establishment of Criteria for Stx1 Operon Identification

The internal identity between the protein sequences of the 12 Stx1a subtypes is higher than 98.7% (Table 1). The internal identity between four Stx1c subtypes’ protein sequences is higher than 98.3%. Only one variant, respectively, of Stx1d and Stx1e has been identified. Amino acid sequences of the complete Stx1 holotoxins are shown in Figure 1. As a result, cutoff values for subtypes were set at 98.3% protein identity for Stx1.

#### 3.1.2. Establishment of Criteria for Stx2 Operon Identification

**Figure 3:**
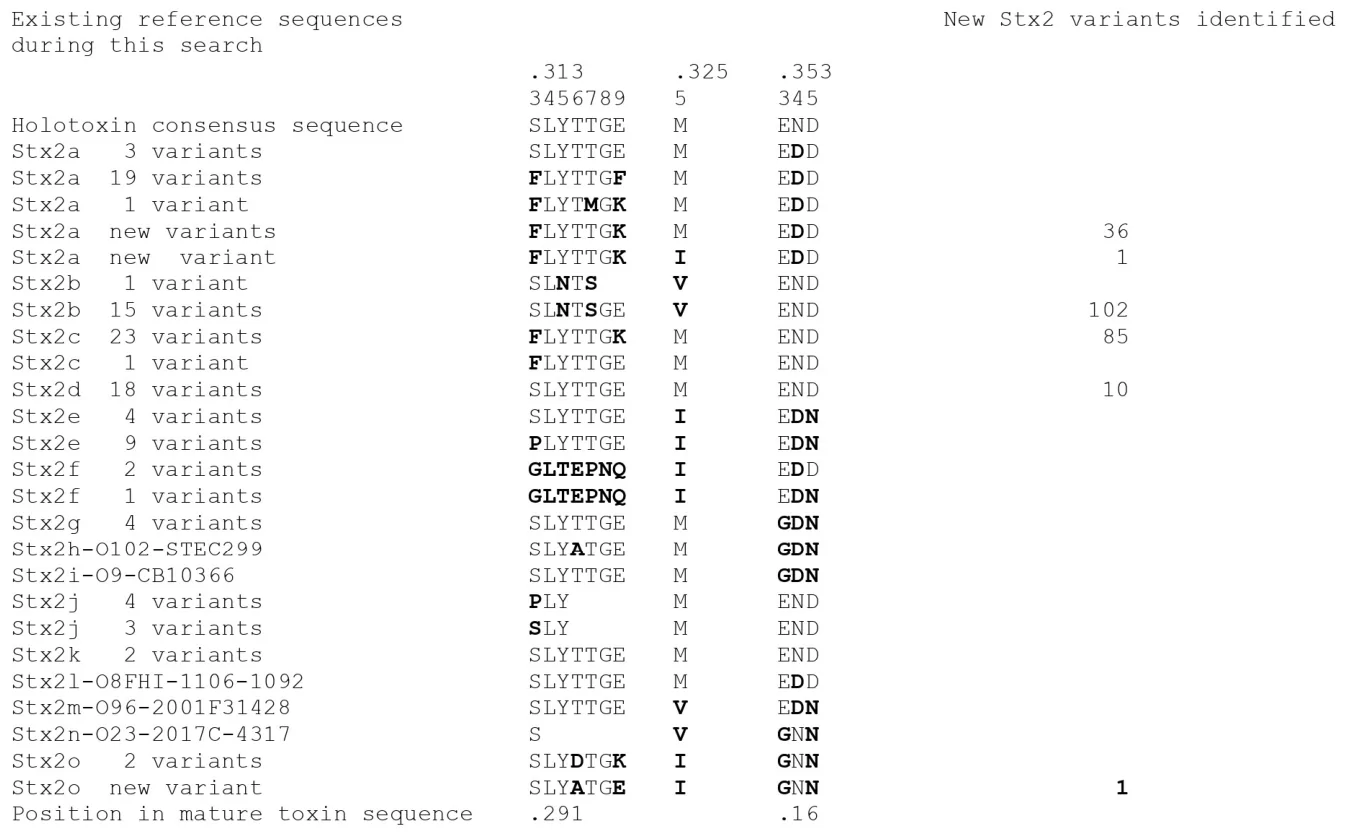
Amino acid sequences of the Stx2 (top numbers correspond to the holotoxin sequence shown in Figure 2) and shows the end of the A subunits in the mature toxins (bottom numbers) of the 117 existing variants within the 15 subtypes of Stx2. The number of new Stx2 variants are shown in the right column. The sequence between serine (S) at position 291 and glutamic acid (E) at position 297 (SLYTTGE) in the mature toxin is the activatable tail. In combination with the END motif at position 16-18 in the mature B subunit this seems to be defining the activatable property of the Stx2d subtype. Amino acid at position 325 may also assist in designating the subtype. These motifs are also found in the two Stx2k subtypes, which however are less than 98.2 identical to Stx2d subtypes. Amino acids that differ from the consensus sequence are shown in bold. Subtypes Stx2g, four Stx2a, five Stx2b, four Stx2e, two Stx2i, Stx2l and Stx2m variants share the SLYTTGE at the tail of the A subunit but differ in their B-subunit.

A sequence alignment of the concatenated Stx2 protein sequences is shown in Figure 3; only single sequences for Stx2h, Stx2m, and Stx2n were identified. The internal protein identity between each of the 15 Stx2 subtypes is higher than 98% (Table 2). Based on the similarities in Table 2 and empirical testing, we used a cutoff of 98% identity for Stx2, except for the very closely related Stx2l and Stx2k (98.5% cutoff) and the cluster of sequences for Stx2a, Stx2c and Stx2d. Given the sequence similarity among types Stx2a, Stx2c, and Stx2d, neither BLAST-based nor HMM-based approaches can distinguish these types. The algorithm described below uses differences at diagnostic sites which allow existing subtype designations to be retained and highlights the significant differences in biological activities and virulence potential among these types. This approach also avoids the introduction of additional confusion to the nomenclature of these cytotoxins. Specific motifs in Stx2 have been related to activation of the toxin, both in vivo and in vitro. The sequence between serine (**S**) at position 313 and glutamic acid (**E**) at position 319 (**SLYTTGE**) in the mature toxin is referred to as the activatable tail (Figure 3). In combination with the END motif at position 16–18 in the mature B subunit, this seems to define the activatable property of the Stx2d subtype and is thus used for Stx2d subtype definition.

### 3.2. Reference Sequences and Reference Collection

Reference strains for each of the subtypes are deposited and available from the European Union Reference Laboratory for *E. coli*, Istituto Superiore di Sanità, Rome, Italy (EURL-VTEC, crl.vtec@iss.it). The individual sequences used by StxTyper to identify genes are listed in Supplementary Table S6 and are available in the Pathogen Detection Reference Gene Catalog (https://www.ncbi.nlm.nih.gov/pathogens/refgene/).

### 3.3. Typing Algorithm

Using the results of this survey, we developed a typing scheme that recapitulates the type determinations described above on novel sequences. This typing scheme is only applicable to complete operon sequences that contain both the StxA and StxB subunits. The algorithm maintains all described types in accordance with recent publications and designations developed at the International Centre for Reference and Research on *Escherichia* and *Klebsiella* [4,5,6,7,18,21,22,23]. In addition, the algorithm identifies sequences that are not part of the existing scheme and could be novel subtypes. The algorithm is as follows:

- First, compute the combined percent identity as the sum of identities of both proteins/sum of reference lengths of both proteins. Both subunits must be full-length, otherwise only the type is called (i.e., Stx1 vs. Stx2).
- A subtype is declared if:
  ○ Both subunits are of the same Stx type, considering Stx2a, Stx2c, and Stx2d as one generalized type “stx2acd”.
  ○ Intergenic region between subunit A and subunit B is <=36 bp.
  ○ The combined percent identity >= the cutoff which is:
    ▪ 98.3% for Stx1 types.
    ▪ 98.5% for Stx2k and Stx2l.
    ▪ 98.0% for the other Stx types.
- If both subunits are full-length and none of the above rules agree to define a sub-type, then it is deemed a novel Stx type (Table 4).

### 3.4. StxTyper Software

We implemented the above Stx operon typing algorithm in the publicly available StxTyper software and database (https://github.com/ncbi/stxtyper). StxTyper not only implements the scheme described above but also detects incomplete or non-functional operons and attempts to provide information about them as well. It identifies Stx operons that do not contain complete sequences of known operon types and attempts to identify whether those operons are partial, contain internal stop codons (i.e., nonsense mutations), extended coding sequences, or frame shifts. The StxTyper software has also been incorporated into the AMR stress resistance and virulence gene identification software, AMRFinderPlus version 4.0 [12] (https://github.com/ncbi/amr), where it is run whenever the --organism Escherichia and --plus options are used and nucleotide input is provided. As part of AMRFinderPlus, StxTyper analysis is included in the Pathogen Detection pipeline and is run on all *E. coli* and *Shigella* genomes as part of normal processing (https://www.ncbi.nlm.nih.gov/pathogens [24]).

### 3.5. Interpreting StxTyper Output

The contents of the “operon” column (called “Method” in AMRFinderPlus) aid in the interpretation of the results by indicating the type of source sequence that was identified. Values of FRAME_SHIFT, INTERNAL_STOP, PARTIAL_CONTIG_END, EXTENDED, or PARTIAL indicate putatively incomplete or non-functional, and therefore not subtyped, operons. Operons that do not meet any of these characteristics are designated as either COMPLETE, COMPLETE_NOVEL, or AMBIGUOUS and, where possible, given the complete operon typing scheme, typed down to the subtype (e.g., Stx2a). Stx operons that are putatively incomplete or non-functional are assigned to type if possible (i.e., Stx1 or Stx2). Thus, all partial or complete Stx operons are assigned one of the following values in the operon field:

- COMPLETE—This operon that can be fully typed to subtype level using the above algorithm.
- PARTIAL—These are partials internal to a contig and, unless there is an error in assembly, likely to not be functional.
- PARTIAL_CONTIG_END—This operon could be a complete operon split across contig boundaries in the assembly, Stx operons are often difficult to assemble, and this could represent a full operon in the source genome that was difficult to sequence and assemble.
- FRAMESHIFT—One (or both) of the subunits has a frame shift detected by StxTyper and is less likely to be a functional operon.
- INTERNAL_STOP—One (or both) of the subunits has an internal stop codon detected by StxTyper.
- COMPLETE_NOVEL—The two subunit genes are fully aligned as described above but do not fit into the typing scheme above, and could represent a novel subtype of Stx. To have a subtype designation assigned, please refer to the **Assignment of New Subtypes** section below.
- AMBIGUOUS—Coding sequences that contain IUPAC ambiguity codes that lead to ambiguities in amino acid translation cannot be fully subtyped. These would otherwise be COMPLETE or COMPLETE_NOVEL.
- All operons with values other than COMPLETE are only resolved to the type level (i.e., Stx1 or Stx2).

### 3.6. Validation Using the Danish Stx Surveillance Set

To validate the algorithm and software, we utilized a set of 2486 surveillance isolates that were sequenced with paired-end Illumina short reads, as part of Danish pathogen surveillance, and submitted to the public Sequence Read Archive. These data were analyzed three ways, and the resulting subtype calls were compared: (i) By using the Danish national typing approach, which integrates assembly-based analysis with the *E. coli* BioNumerics plugin and complementary read-mapping approaches. In cases of ambiguous results, subtypes were resolved through manual curation supported by supplementary mapping analyses or confirmatory PCR assays [16,18].

(ii) The same WGS sequences were also assembled and analyzed by the NCBI Pathogen Detection pipeline including StxTyper and submitted to GenBank. (iii) The NCBI Pathogen Detection assemblies in GenBank were separately analyzed with VirulenceFinder (Supplementary Table S1). Note that the NCBI Pathogen Detection pipeline uses two assemblers, the de novo assembler SKESA [25] and, for added sensitivity and discriminatory power, appends a SAUTE guided assembly [26] using guide sequences from AMR reference genes and reference Stx operons. This leads to high sensitivity but also means that contigs containing sequences related to the guide sequences (including Stx operons) are often duplicated in the final assembly; therefore, in the analyses described below, we de-duplicated Stx operon calls by subtype; so for a given isolate, each subtype is only represented once no matter how many times it appears in the assembly.

#### 3.6.1. Comparison to the Danish Surveillance Stx Typing Protocol

Of the 2486 isolates in this test set, 2452 isolates (98.6%) had identical calls, and 34 isolates (1.4%) had different calls between the Danish surveillance method and StxTyper ran on Pathogen Detection assemblies (Supplementary Table S1). StxTyper on the Pathogen Detection assemblies identified 24 complete operon subtypes that were not found with the Danish surveillance method in 18 assemblies (0.7%). Conversely, the Danish method identified a Stx subtype in eight isolates (0.4%), whereas none were found with StxTyper in NCBI assemblies. On an operon basis, the Danish method identified 20 operon subtypes in 19 isolates (0.76%) that were not called by StxTyper in NCBI Pathogen Detection assemblies. Looking just at types instead of subtypes (i.e., Stx1 vs. Stx2) in all cases but one, the two methods agreed on the Stx types identified for each isolate.

We took a closer look at the assemblies for the 19 isolates where Danish surveillance methods identified Stx subtypes that were not identified with the NCBI Pathogen Detection assemblies and StxTyper. These 19 discrepancies could be explained by the absence of full-length operons in the NCBI Pathogen Detection assemblies. To understand if this combination of assemblers used by NCBI Pathogen Detection missed information in the reads, we aligned reads to the reference sequences for the expected Stx types. In all cases, the alignment had areas of no or low (<4x) read coverage in parts of the operon. Areas of low or no-coverage will cause breaks in the assembly, and were likely responsible for the lack of full-length operons in the resulting assembly [25,26]. This is a reminder of the importance of sequence quality, coverage, and representativeness for sensitive and accurate detection of Stx operons from whole-genome shotgun assembly.

#### 3.6.2. Comparison to VirulenceFinder Results on NCBI Pathogen Detection Assemblies

To prevent the effects of assembly from obscuring differences in sequence typing methods, we compared the results from the VirulenceFinder database used in the Danish surveillance method, but applied to the same NCBI Pathogen Detection assemblies analyzed with the StxTyper above. Of the 34 isolates that had differences between the Danish surveillance method and StxTyper described in the previous section, 15 (44%) of those differences were resolved when comparing the VirulenceFinder analysis on identical assemblies (Supplementary Table S1).

In total, 146 of the 2486 isolates (6%) had differences in calls between StxTyper and VirulenceFinder on these identical assemblies. In those isolates, VirulenceFinder made 148 Stx operon subtype calls that were not made with StxTyper; however, all those operon calls were for operons identified by StxTyper but assigned a different subtype. The differences in subtype call were explained by three differences between StxTyper and VirulenceFinder: (i) StxTyper does not subtype operons that are detected as PARTIAL, PARTIAL_CONTIG_END, FRAMESHIFT, or INTERNAL_STOP, whereas VirulenceFinder will call a subtype for anything meeting its minimum nucleotide blast cutoffs (60% coverage and 90% identity). (ii) StxTyper uses a diagnostic-site algorithm to differentiate the very closely related Stx2a, Stx2c, and Stx2d operons; here, 17 Stx2c operons were identified as other subtypes by VirulenceFinder because it uses the nearest nucleotide blast hit rather than the amino acid sequence and diagnostic sites. (iii) VirulenceFinder may call multiple equally distant hits. In the Danish surveillance typing method, these are resolved by manual alignment and examination. Here, we used the raw output of VirulenceFinder without the additional alignment and manual examination.

StxTyper called five Stx subtypes that were not called by VirulenceFinder. Two were the *Shigella sonnei* Stx1 which StxTyper calls Stx1a, but VirulenceFinder does not subtype. The remaining three were Stx2a and Stx2c which are very closely related and differentiated by diagnostic amino acid sites. Differentiation of Stx2a and Stx2c with VirulenceFinder relies on nucleotide similarity to database entries and may be incorrect for nucleotide sequences not included in its database due to synonymous mutation. Overall, these results demonstrate that VirulenceFinder and StxTyper largely make the same call on full-length operons, though there are rare cases where the VirulenceFinder call may be incorrect or where manual examination of VirulenceFinder results is necessary.

### 3.7. Comparison to California Department of Public Health PCR Results

The California Department of Public Health sequenced and characterized 523 isolates as part of their routine STEC surveillance. Prior to entering the sequencing workflow, a real-time PCR (RT-PCR) assay to detect and differentiate Stx1 and Stx2 operons was performed [20]. We compared the PCR results for those isolates to the results from NCBI Pathogen Detection and found a high degree of correlation (Supplementary Table S2). For 520 of the 523 isolates (99%), StxTyper and the RT-PCR methods identified the same Stx operon types. Both PCR and sequencing were repeated for samples with discrepant results. For two of the three isolates that were called differently, StxTyper detected complete Stx1 operons that were not identified by PCR. It is possible that compounds in the bacterial lysate inhibited the PCR reaction and Stx detection [27]. For the third, the PCR assay identified both Stx1 and Stx2 operons, but StxTyper identified only Stx2 operons; a search of the assembly and reads found no evidence of an Stx1 operon in that assembly or the reads indicating the sequencing run did not contain any data that would explain the PCR results. Potential explanations for the discrepant results include PCR amplification of cross-reacting sequence, gene loss due to bacteriophage instability, or limitation of short-reading sequencing [28,29].

### 3.8. StxTyper Analysis of Stx-Gene-Containing Isolates in NCBI Pathogen Detection

The NCBI Pathogen Detection System contains a large number of *E. coli* and *Shigella* assemblies generated by the Pathogen Detection system, as described above, based on short-read sequences submitted by public health agencies, researchers, hospitals, and others, as well as other assemblies generated by submitters to GenBank with various sequencing and assembly methods. Here, we summarize the results of running StxTyper on that full list of assemblies available in NCBI Pathogen Detection [24].

We used StxTyper to screen all 371,996 *E. coli* and *Shigella* in NCBI’s Pathogen Detection with accessioned genomes in GenBank as of 3 April 2025; this set includes all the previously described assemblies. A total of 111,781 of those isolates (30%) had complete or partial operons detectable with StxTyper, and 108,985 (97.5%) had at least one complete Stx operon in the assembled sequence (Supplementary Table S7). Stx1a and Stx2a were the most common subtypes in 57% and 36% of isolates with typable Stx operons (Table 5). Note that this set consists of publicly deposited isolate sequences from *E. coli*, *E. albertii*, and *Shigella*; it is not a surveillance study, and selection bias is present. These results will not be representative of a specific population (e.g., [30]) or other species with Stx operons such as *E. marmotae*.

#### Combinations of Operons

A total of 34,580 isolates (32% of isolates with complete operons) had multiple operons of different types, and 934 isolates (0.86%) had three or more Stx operons of different subtypes (Supplementary Table S8). The most common combinations of Stx operons were Stx1a and Stx2a (11%), Stx2a and Stx2c (9.6%), and Stx1a and Stx2c (4.9%). This is consistent with Stx1a, Stx2a, and Stx2c being the three most common Stx subtypes in this set (Table 5). This high frequency of isolates with multiple Stx operons is likely the result of multiple phage insertions and has been noted before, but on smaller numbers of isolates and with significantly different frequencies of combinations of subtypes (e.g., [10,31,32]).

## 4. Discussion

In this paper, we present a survey of known Stx operon sequences, including all known subtypes. We used that survey to develop a concrete algorithm to accurately type Stx operons. We then used that algorithm to develop the software, StxTyper, to accurately identify subtypes as well as to determine operons for which the scheme cannot be fully applied to identify subtypes. We compared the results of running StxTyper on assembled short-read sequences to Stx typing information provided by two public health surveillance systems using alternative methods. One dataset is from Denmark, using a combination of PCR-based and whole-genome-shotgun (WGS) sequence-based methods for Stx subtyping, and one is from the California Department of Public Health, using a real-time PCR-based method for typing, both showing a high degree of concordance. We further applied StxTyper to very-large-scale data available in the NCBI Pathogen Detection System, identifying the most common patterns of Stx operons in over 371,000 publicly available *E. coli*, *E. albertii*, and *Shigella* genomes.

Traditionally, Stx operon types lacked a formal sequence-based definition, with experts resolving edge cases on the basis of the manual inspection of alignments. This generates several barriers to using automated systems to correctly identify Stx subtypes in high-throughput pipelines and, importantly, to identify when those automatically determined types are unclear or novel. The scheme developed and introduced in this paper provides a formalized approach allowing the consistent and accurate typing of all currently described Stx types, from complete operon sequence to the identification of potentially novel Stx operon types. StxTyper also identifies operons with lesions that make them incomplete or otherwise untypable with this scheme. The identification of operons that do not fit within this scheme is particularly relevant given the frequent assembly errors or breaks in Stx operon regions from contigs assembled from short-read whole-genome-shotgun (WGS) data.

Using this algorithm and the publicly available open-source software StxTyper, the NCBI Pathogen Detection System is able to provide near real-time type information for the over 371,000 publicly available *E. coli* and *Shigella* isolate assemblies in NCBI Pathogen Detection, with new isolates added almost daily. Untypable operons may be marked as COMPLETE_NOVEL or PARTIAL or any of the other categories, and in some cases may represent thus far undescribed Stx subtypes.

### Assignment of New Stx Subtypes

Recently, several new subtypes of Stx have been identified and characterized (e.g., [4,5]); the increasing rate of whole-genome sequencing leads to an increasing likelihood of multiple groups identifying novel subtypes and causing naming collisions. To avoid possible collisions in subtype naming for novel subtypes and to maintain agreement on subtype designations, we propose that NCBI, in consultation and collaboration with EURL-VTEC and outside experts, assign new Shiga toxin subtypes. Subtype assignments will be only assigned for naturally occurring operons with amino acid sequences for both StxA and StxB subunits that do not fit into the existing typing scheme, which are sufficiently phylogenetically or functionally different from existing named subtypes, and where phylogenetic and functional studies of sequences and phenotypic analyses, including assessment of expression and toxicity, are performed.

Preliminary assignment would require: (i) agreement that the sequence represents a novel subtype and is not a new member of an existing subtype, (ii) the full-length nucleotide sequence of the Stx operon submitted to GenBank or made publicly available in International Nucleotide Sequence Database Collaboration (INSDC) databases, including annotation of the A and B subunits in consultation with NCBI and outside experts, (iii) the deposition of a reference strain with the European Union Reference Laboratory for *E. coli* (EURL-VTEC), (iv) functional characterization work should be planned or ongoing and should include Vero cell cytotoxicity assays, and (v) follow the instructions at https://www.ncbi.nlm.nih.gov/pathogens/stx/ to contact NCBI and submit the subtype assignment request. If submitters are unclear as to whether they have a new subtype, they should contact NCBI at pd-help@ncbi.nlm.nih.gov. While curators will make provisional assignments before characterization work is completed, full assignment requires the completion and validation of function by EURL-VTEC on the provided reference strains. All updates to and modifications of the typing scheme will be announced in a new release of StxTyper.

## Figures and Tables

**Table 1:** **Amino acid**/*nucleotide identities* (%) using Neighbor Joining comparison in BioNumerics version 8.1 (Applied Maths, Biomérieux), within and between 18 Stx1 subtypes and variants, accessions listed in Supplementary Table S5.

	*stx1a*	*stx1c*	*stx1d*	*stx1e*
Stx1a	**98.7**/*98.5*	*95.7–97*	*91.3–91.9*	*85.9–86.3*
Stx1c	**96.8–98.3**	**98.3**/*99.1*	*92.3–92.6*	*86.6–87.1*
Stx1d	**95.4–96.1**	**95–96**	**100**	*86.8*
Stx1e	**91.1–91.6**	**91.1–92.1**	**91.9**	**100**

**Table 2:** Amino acid identities (%) using Neighbor Joining comparison in BioNumerics version 8.1 (Applied Maths, Biomérieux), within (minimum) and between (maximum) 117 Stx2 subtypes. Identities in bold indicate identities between subtypes that are higher than the within identities.

	Stx2a	Stx2b	Stx2c	Stx2d	Stx2e	Stx2f	Stx2g	Stx2h	Stx2i	Stx2j	Stx2k	Stx2l	Stx2m	Stx2n	Stx2o
Stx2a	**98.8**														
Stx2b	96.8	**98.1**													
Stx2c	**99.9**	96.7	**98.7**												
Stx2d	**99.7**	97.6	**99.7**	**98.6**											
Stx2e	95.9	95.0	95.6	96.1	**98.2**										
Stx2f	82.3	81.4	82.2	82.4	84.1	**98.8**									
Stx2g	97.5	97.4	96.9	97.4	96.0	82.4	**98.9**								
Stx2h	95.7	95.9	95.5	95.9	94.6	81.8	95.5	N/A							
Stx2i	96.4	94.3	95.9	96.4	97.9	82.4	96.5	95.5	**99.7**						
Stx2j	94.2	93.6	94.5	94.7	93.0	83.2	93.0	94.1	93.0	**98.5**					
Stx2k	97.7	96.0	97.8	98.2	97.2	82.3	96.8	95.9	97.9	94.3	**99.9**				
Stx2l	97.4	94.7	96.8	97.5	97.6	82.8	96.2	94.7	97.9	94.1	98.1	**99.7**			
Stx2m	96.3	95.2	95.6	96.1	94.4	82.4	96.1	94.8	94.5	92.3	95.0	93.8	N/A		
Stx2n	93.6	93.3	95.5	96.0	92.7	84.0	93.5	95.0	93.2	91.8	93.9	92.4	93.2	N/A	
Stx2o	94.9	94.5	95.3	95.3	94.0	82.0	94.9	97.1	94.7	93.2	95.5	94.1	93.8	94.8	**99.7**

**Table 3:** Diagnostic positions in the stx2 holotype alignment.

Stx Subtype	313	319	325	353	354	355
Stx2a	F/S	K/E	M	E	D	D
Stx2b	S	K/E/-	V	E	N	D
Stx2c	F	K/E	M	E	N	D
Stx2d	S	E	M	E	N	D
Stx2e	P/S	E	I	E	D	N

Using this algorithm, we identified 37 new Stx2a, 102 Stx2b, 85 Stx2c, ten Stx2d and one Stx2o variants, shown in Figure 3.

**Table 4:** Sites used to differentiate Stx2a, Stx2c, and Stx2d.

Stx Subype	Subunit A		Subunit B
Position	313	319	35 (354 in Holotoxin Alignment)
Stx2a	F/S	K/E	D
Stx2c	F	K/E	N
Stx2d	S	E	N

This table determines the exact S type for the generalized “Stx2acd” type; if an amino acid is present at those locations not on the chart, then it may be called Stx2, but is a novel subtype.

**Table 5:** Count of assemblies with complete operons for each Stx type for the Pathogen Detection dataset.

Stx Type	Number of Isolates
stx1a	61,836
stx2a	39,687
stx2c	21,903
stx2b	6739
stx1c	6441
stx2d	3517
stx2e	1529
stx2f	1057
stx2g	483
stx2j	388
stx1d	305
stx2k	271
stx2n	144
stx2l	88
stx2i	81
stx2*	15
stx2o	11
stx2h	7
stx2m	6
stx1 *	2

* operons that are full length, but are not assigned a subtype by the typing scheme. These may be novel Stx types that have not been previously described but await characterization to be added to the system.

## Data Availability

All sequence data are deposited in INSDC repositories; accessions are provided in Supplementary Tables S1–S3 and S6. Results of alternative typing methods are provided in Supplementary Tables S1 and S2.

## Supplementary Materials

The following supporting information can be downloaded at: https://www.mdpi.com/article/10.3390/microorganisms14081607/s1, Supplementary Table S1: Danish surveillance isolates and Stx type calls. Supplementary Table S2: Real-time PCR dataset from the California Department of Public Health. Supplementary Table S3: 111,781 isolates in Pathogen Detection MicroBIGG-E with Stx genes. Supplementary Table S4: StxTyper results for 111,781 isolates in Pathogen Detection MicroBIGG-E with Stx genes. Supplementary Table S5: Stx operon sequences analyzed to develop typing scheme. Supplementary Table S6: Reference Stx gene sequences used by StxTyper. Supplementary Table S7: “Operon” column value counts for 111,781 isolates in Path. Supplementary Table S8: Combinations of Stx operon types.
